# Supplementary material for: Antioxidants and Therapeutic Targets in Ovarian Clear Cell Carcinoma
Source: Antioxidants (Basel). 2021 Jan 28;10(2):187. doi: 10.3390/antiox10020187 (PMC7911626; doi:10.3390/antiox10020187)
Supplement: Supplementary file 1 [file antioxidants-10-00187-s001.pdf]

Supplementary Materials

Supplementary Table 1. Upregulated 79 genes in vividly growing cancer spheres.

Up-Gene Symbol	Gene ID	Fold change*	*p*-values**
LITAF	9516	2.1207592	2.03E-04
EFTUD1	79631	2.1516137	3.35E-04
SMOC1	64093	4.4188356	8.81E-04
CLDN24	100132463	3.3124933	9.66E-04
MGLL	11343	2.2893085	9.91E-04
SEPW1	6415	2.4701314	0.001132938
C11orf96	387763	2.1179924	0.001323551
PIGQ	9091	2.9436288	0.001981718
MIR21	406991	4.4390764	0.002264138
C19orf71	100128569	5.2451873	0.002283707
ARRDC4	91947	4.73275	0.002435295
ANKRD9	122416	2.7407963	0.002756929
ARNT2	9915	2.3692644	0.003850413
FIBCD1	84929	3.6863317	0.003900672
MIR645	693230	2.7949657	0.00419125
ZNHIT2	741	2.685165	0.004256217
FLJ46906	441172	2.511955	0.004829411
TP53INP2	58476	2.2619126	0.005131293
UBE2Z	65264	2.0384877	0.005149033
MSTO2P	100129405	2.575711	0.005210416
RAB33A	9363	2.091018	0.005354992
PDXP	57026	2.1576443	0.005404675
CTRL	1506	2.0714319	0.005804448
MOCOS	55034	2.1209774	0.006158605
G0S2	50486	11.819523	0.006494332
SNORA53	677832	2.264417	0.006705817
REPIN1	29803	2.8456938	0.006773508
SNORD83A	116937	3.1943386	0.006857268
SGK223	157285	2.1261873	0.007217159
INO80B	83444	2.150224	0.007590249
FAM101B	359845	2.0070763	0.00805826
MICAL2	9645	2.149102	0.008159041
DEXI	28955	2.4966269	0.008739237
CCNG2	901	2.0922632	0.008928092
SH2D2A	9047	4.580897	0.009170096
SNORA57	692158	2.170166	0.009371574
CAPN10	11132	2.1131349	0.010219667
YPEL3	83719	3.6506276	0.010413257
SIRPA	140885	2.242258	0.010780468
AMT	275	2.1465266	0.011944363
UBALD2	283991	2.6380594	0.011965454
TBC1D2B	23102	2.142051	0.013069578
MIR573	693158	2.6520598	0.013145302
CD81	975	2.4236543	0.013877614
BHLHE41	79365	2.3765693	0.014037296
FOXP4	116113	2.1288257	0.015122701
SLC8B1	80024	2.6130712	0.01517499
LIN7A	8825	2.4526713	0.015736822
TRIM8	81603	2.2736151	0.015909232
METTL24	728464	3.7812667	0.016054606
ATP6V1B1	525	2.3591247	0.016124561
C2CD4A	145741	2.9770472	0.016409228

RPL23AP82	284942	2.0265024	0.01709264
RRAS	6237	3.475575	0.017852157
GPC2	221914	2.199905	0.017908165
ALDH1A3	220	2.1348593	0.018868158
FURIN	5045	2.3936615	0.019097326
ANKRD20A1	84210	3.9995637	0.01910931
BBC3	27113	2.191234	0.019140063
ATP13A2	23400	2.2020829	0.019162841
TMEM8A	58986	2.692243	0.019301366
TTLL3	26140	2.0526586	0.019753855
SNORD49A	26800	2.5610104	0.020195134
HOXD9	3235	2.687174	0.021076625
ZNF513	130557	3.8625216	0.022652812
PRR14	78994	2.6562395	0.02285078
HSFX2	100130086	2.97543	0.023185235
TPRA1	131601	2.0928478	0.02320262
CXCL3	2921	6.3271894	0.023270097
RDH10	157506	2.6080832	0.0239462
CPNE2	221184	2.837128	0.024443708
ABHD17C	58489	4.298869	0.024986247
JARID2-AS1	100506681	2.3300421	0.025002286
SSSCA1	10534	2.2366126	0.025128437
APRT	353	2.3234353	0.025269138
VWA5A	4013	2.4164116	0.025834825
HIC1	3090	2.1755214	0.026201908
LAMB3	3914	2.866365	0.026381055
MAMLD1	10046	2.3176277	0.02675306

*These genes were statistically upregulated in vividly growing spheres irrespective of continuously RAB39A-knockdown.

**Replicate analysis (Strand NGS); Moderate T-test followed by Westfall Young correction was performed between vividly growing spheres and poor ones.
